# Impact of pre-hospital handling and initial time to cranial computed tomography on outcome in aneurysmal subarachnoid hemorrhage patients with out-of-hospital sudden cardiac arrest—a retrospective bi-centric study

**DOI:** 10.3389/fcvm.2023.1209939

**Published:** 2023-08-21

**Authors:** Tobias Pantel, Axel Neulen, Marius Marc-Daniel Mader, Elena Kurz, Andras Piffko, Verena Fassl, Manfred Westphal, Jens Gempt, Florian Ringel, Patrick Czorlich

**Affiliations:** ^1^ Department of Neurosurgery, University Medical Center Hamburg-Eppendorf, Hamburg, Germany; ^2^ Department of Neurosurgery, University Medical Center of the Johannes Gutenberg-University Mainz, Mainz, Germany

**Keywords:** subarachnoid hemorrhage, sudden cardiac arrest, resuscitation, emergency room handling, lay cardiopulmonary resuscitation, CPR

## Abstract

**Background:**

Aneurysmal subarachnoid hemorrhage (SAH) presents occasionally with cardiac arrest (CA). The impact of pre-hospital and emergency room (ER) treatment on outcome remains unclear. Therefore, we investigated the impact of pre-hospital treatment, focusing on lay cardiopulmonary resuscitation (CPR), and ER handling on the outcome of SAH patients with out-of-hospital CA (OHCA).

**Methods:**

In this bi-centric retrospective analysis, we reviewed SAH databases for OHCA and CPR from January 2011 to June 2021. Patients were analyzed for general clinical and epidemiological parameters. CPR data were obtained from ambulance reports and information on ER handling from the medical records. Data were correlated with patient survival at hospital discharge as a predefined outcome parameter.

**Results:**

Of 1,120 patients with SAH, 45 (4.0%) were identified with OHCA and CPR, 38 of whom provided all required information and were included in this study. Time to resuscitation was significantly shorter with lay resuscitation (5.3 ± 5.2 min vs. 0.3 ± 1.2 min, *p* = 0.003). Nineteen patients were not initially scheduled for cranial computed tomography (CCT), resulting in a significantly longer time interval to first CCT (mean ± SD: 154 ± 217 min vs. 40 ± 23 min; *p* < 0.001). Overall survival to discharge was 31.6%. Pre-hospital lay CPR was not associated with higher survival (*p* = 0.632). However, we observed a shorter time to first CCT in surviving patients (*p* = 0.065)

**Conclusions:**

OHCA in SAH patients is not uncommon. Besides high-quality CPR, time to diagnosis of SAH appears to play an important role. We therefore recommend considering CCT diagnostics as part of the diagnostic algorithm in patients with OHCA.

## Introduction

Aneurysmal subarachnoid hemorrhage (SAH) is a devastating type of hemorrhagic stroke, associated with relevant short- and long-term morbidity and mortality (1–4). In addition to acute neurological symptoms, complications in other organ systems may occur at the moment of aneurysm rupture (5). Over the last few decades growing evidence of impaired cardiac function in the acute phase of SAH has emerged (6, 7). In current studies, the cause is not primarily seen in a cardiac problem but rather in a dysregulation of the neuro-cardiac axis (5–11). In a subset of SAH patients, this leads to neurogenic stress cardiomyopathy (NSC), which, depending on severity, can cause impaired cardiac function and thereby contribute to cerebral hypoperfusion (6, 7, 9, 11). In addition, it is widely accepted that SAH is one of the most relevant non-cardiac causes of sudden cardiac arrest (CA) (8, 12). Although there have been several studies focusing on this, the rate of occurrence remains unclear and ranges from 3% to 11%, which is most likely due to the fact that an unknown number of patients die before hospital admission (13, 14).

Many studies have demonstrated the critical role and the positive impact of immediate resuscitation on patient’s survival, independent of the underlying disease (15–17). Patients who were resuscitated immediately were reported to have a higher overall survival compared to those who were not resuscitated immediately (6). For SAH patients with out-of-hospital CA (OHCA), however, data on the impact of immediate initiation of lay cardiopulmonary resuscitation (CPR) are missing.

In case of return of spontaneous circulation (ROSC), rapid transport of the patient to a hospital is necessary for further diagnostics and treatment of the underlying cause (18, 19). In patients with post-resuscitation conditions, the focus of emergency diagnostics is primarily on cardiac and internal diseases, which are statistically the most likely underlying cause of CA (19). However, this can lead to neurological causes such as SAH being considered only subsequently, and the patient is therefore transferred to cranial computed tomography (CCT) imaging and further therapy with delay. A negative influence on the outcome is likely, as the importance of an optimal emergency room (ER) management and rapid diagnosis has been demonstrated for SAH patients (20). However, data on this aspect are missing.

Hence, in this study, we set out to investigate both the impact of pre-hospital treatment, focusing on lay CPR, and the ER management in patients with cardiac arrest and SAH with regard to survival and neurological outcome.

## Materials and methods

### Patient data

The study was approved by the responsible ethics committees [local ethical review board of Hamburg, Germany (WF-010/21) and Ethics Committee of the Rhineland-Palatinate Medical Association (2021–15968)] and was performed in accordance with the ethical standards laid down in the Declaration of Helsinki and its later amendments. Since the data were anonymized, and the study was retrospective, informed consent was waived.

### Data acquisition

An analysis of all patients treated for SAH in both tertiary medical centers from January 2011 to June 2021 was performed. The aneurysmal nature of SAH was verified by cerebral digital subtraction angiography (DSA)/cranial computed tomography angiography (CTA). Radiologically proven aneurysms were classified according to their location in the anterior or posterior circulation. All patients were further evaluated with respect to OHCA, which was the inclusion criterion for this study. Patients with unclear information on the CA or with in-hospital CA (IHCA) were excluded. Focus was on prognostic data including the Glasgow Coma Scale (GCS), World Federations of Neurosurgical Societies (WFNS) scale, and Fisher grade, which were gathered from patients’ health records. Since patients with CA generally demonstrated a GCS of 3, all patients in this study were classified as WFNS 5 accordingly. In addition, the cardiac rhythm at time of the first electrocardiogram (ECG) was obtained (see Table 1).

**Table 1:** Population characteristics for patients with SAH and OHCA.

Feature	OHCA (*n* = 38)
Age (years), mean (SD)	55.1 (13.9)
Female, *n* (%)	20 (52.6)
First documented cardiac rhythm	
Asystole	14 (36.7)
Ventricular fibrillation	2 (5.3)
Pulseless electrical activity	11 (29)
ROSC/unknown	11 (29)
Fisher score, *n* (%)	
3	3 (7.9)
4	35 (92.1)
Aneurysm location, *n* (%)	
Anterior circulation	21 (55.3)
Posterior circulation	17 (44.7)
Aneurysm multiplicity, *n* (%)	5 (13.2)
Aneurysm diameter (mm), mean (SD)	10 (7.6)

Patients’ emergency protocols were screened with respect to CPR information, such as CPR type, duration to initiation of CPR, initial cardiac rhythm, and duration to ROSC. In the case of lay CPR, the duration until the start of CPR was assumed to be immediate (time value set as 0 min if detailed information on time period was missing). In cases of professional resuscitation, the time from the receipt of the emergency call to the arrival of the emergency service was used, which was extracted from the emergency service protocols. Information on the initial ER management of each patient after ROSC and hospital admission were extracted from the patients’ health records. Focus was on the initially assigned department and the duration from hospital admission to first CCT scan. The treatment for the SAH patients in our study was performed in specialized intensive care units in accordance with the current international guidelines as well as institutional treatment protocols (21, 22).

All included patients were evaluated for short-term outcome, which was defined as in-hospital mortality. The modified Rankin scale (mRs) was used to assess the neurological status of these patients at discharge.

### Statistical analysis

Data were extracted from the electronic patient records and transferred to anonymized data tables. Data were collected at both centers by one investigator each. To minimize errors in the collected data, individual data sets were randomly checked by a second investigator. Data are displayed as mean ± SD for continuous variables or absolute and relative numbers for categorical variables. Differences in continuous variables were analyzed with the Mann–Whitney *U* test, and differences in proportions were analyzed with the chi-square test or Fisher’s exact test. A two-sided *p*-value less than 0.05 was considered statistically significant. All analyses were performed using SPSS Statistics Version 29 (SPSS Inc., Chicago, IL, USA).

## Results

### Patient cohort and general characteristics

**Figure 1:**
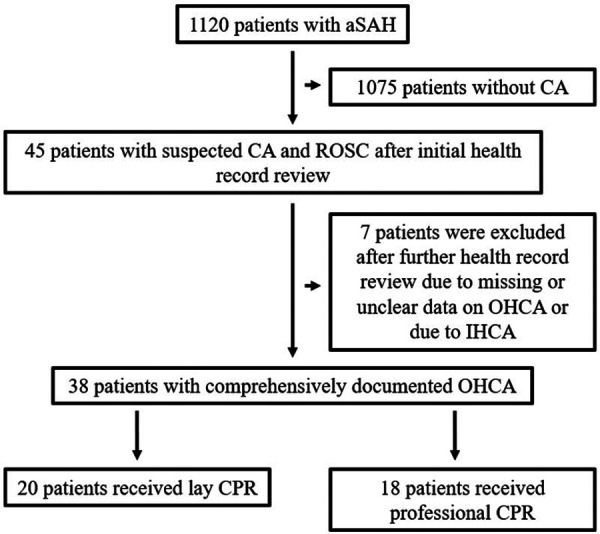
Overview of patient cohort and reasons for exclusion of individual patients. A total of 38 patients with comprehensively documented OHCA were included in the study, 20 of whom were resuscitated by laypersons.

Database analysis identified a total of 1,120 patients treated for aneurysmal SAH at our tertiary medical centers between January 2011 and June 2021. In 45 of these patients, OHCA with CPR was present, resulting in an incidence of 4.0%. Of these, complete data, as described above, were available for 38 patients, which were included in this study (Figure 1). The gender distribution of patients was homogeneous (female = 20, male = 18), and the age of affected patients was 55.1 ± 13.9 years. SAH was classified Fisher 4 in 92.1%. WFNS grade was 5 in all finally included patients. In our SAH cohort, diagnosed aneurysms were over-proportionately localized in the posterior circulation (*n* = 17, 44.7%). In the overall cohort, the aneurysm distribution ratio for the anterior and posterior circulation was 84.3% vs. 15.7%. The clinical characteristics of the patient cohort are shown in Table 1.

### Resuscitation characteristics

Lay CPR was performed in 20 cases (52.6%), and the remaining 18 patients (47.4%) were not resuscitated until arrival of the emergency service. The time taken to start CPR was significantly prolonged for patients first treated by the ambulance service (5.3 ± 5.2 min vs. 0.3 ± 1.2 min, *p* = 0.003). In contrast, the time to ROSC after the start of resuscitation in these patients was 7.4 ± 6.0 min, which was unexpectedly shorter than in patients resuscitated by laypersons (12.4 ± 8.8 min, *p* = 0.054). Focusing on the patients who survived OHCA initially and were hospitalized, there was no statistically significant difference in the mode of resuscitation [20 (lay CPR) vs. 18 (professional CPR), *p* = 0.734]. The majority of patients (65.7%) had non-cardiovertible cardiac rhythms [asystole or pulseless electrical activity (PEA)] at the time of the first ECG. Five patients already had ROSC at this time, and in another six patients, no reliable statement about the initial cardiac rhythm could be made from the records (see Table 1). Overall, 26 (68.4%) patients died during the hospital stay. Compared to survivors (*n* = 12, 31.6%), deceased patients were significantly older [58.4 ± 13.1 years (non-survivors) vs. 48.1 ± 14.7 years (survivors), *p* = 0.048)]. No difference between these two groups was detectable in terms of prognostic factors (e.g., Fisher grade, WFNS score). The time to onset of CPR was without significant difference for both groups, but the time to ROSC was significantly prolonged for the patients who died during the hospital stay [5.0 ± 4.5 min (survivor) vs. 11.6 ± 8.1 min (non-survivor); *p* = 0.013]. A detailed overview is presented in Table 2.

**Table 2:** Resuscitation features and emergency room handling for patients who survived OHCA and those who did not.

Feature	Survivor (*n* = 12)	Non-survivor (*n* = 12)	*p*-value
Age (years), mean (SD)	48.1 (14.7)	58.4 (13.1)	0.048
Fisher score, *n* (%)			
3	1 (8.3)	2 (7.7)	
4	11 (91.7)	24 (92.3)	
Type of CPR, *n* (%)			
Lay CPR	7 (58.3)	13 (50)	0.734
Professional CPR	5 (41.7)	13 (50)	
Time to CPR (min), mean (SD)	0.7 (2.1)	3.2 (4.8)	0.193
Time to ROSC (min), mean (SD)	5.0 (4.5)	11.6 (8.1)	0.013
Emergency room handling in presence of a neurologist/neurosurgeon, *n* (%)	8 (66.7)	15 (57.7)	0.163
Time to CCT (min), mean (SD)	45.7 (35.0)	107.6 (176.7)	0.065
mRS at discharge^a^, *n* (%)			
0	0 (0)	0 (0)	
1	2 (20)	0 (0)	
2	0 (0)	0 (0)	
3	2 (20)	0 (0)	
4	3 (30)	0 (0)	
5	3 (30)	0 (0)	
6	0 (0)	26 (100)	

^a^ Information on neurological status at discharge were missing in two cases, resulting in an overall patient number of 36 cases for this part.

In 19 (50.0%) of the patients, a neurological cause was suspected, and a CCT scan was initiated early during ER treatment. The time to first CCT scan from hospital admission in these patients was 40.2 ± 22.8 min. Conversely, in 19 (50.0%) patients, a neurological cause was initially not suspected. The time to first CCT scan in these patients was significantly longer (154.4 ± 216.6 min; *p* < 0.001). A difference in time to first CCT can further be demonstrated when patients are categorized into survivors and non-survivors. Importantly, survivors demonstrated a shorter interval to first CCT compared to non-survivors (45.7 ± 35.0 min vs. 107.6 ± 176.7 min, *p* = 0.065, Table 2).

## Discussion

In our bi-centric study, we investigated the impact of pre-hospital treatment, focusing on lay resuscitation, as well as initial emergency room handling on the outcome of patients with aneurysmal subarachnoid hemorrhage with OHCA. The key results of our study include the following: (1) SAH is a relevant cause of OHCA. (2) In case of sudden OHCA due to SAH, lay resuscitation was performed in more than half of the cases, without achieving a significant advantage in outcome. In addition, (3) there was a strong trend for better outcome in patients who obtained a CCT scan early.

Over a 10-year period, we treated a total of 1,120 patients with SAH in our neurosurgical departments, of which 45 patients (4%) had experienced OHCA. A similar incidence is also reported in a recent registry study (23) and in other studies (8, 13, 24). In this context, it should be mentioned that in our OHCA cohort, there was no gender dominance and patients were affected in middle age. This is interesting for two reasons: on one hand, a female gender dominance (25) is described in other SAH studies, and, on the other hand, older persons dominate in works on cardiovascular disease (26).

The exact etiology of CA in the acute phase of SAH remains unclear (10, 27, 28). A possible and often discussed mechanism is central apnea, which occurs within the moment of aneurysm rupture and subsequently leads to hypoxia with following brady- or apnea. The next common theory is a central dysregulation due to the rapid increase in intracranial pressure at aneurysm rupture, which leads to a transient global cerebral ischemia (27). Appropriately, in our cohort, PEA and ventricular fibrillation (VF) are the most commonly described initial cardiac rhythms, which confirm the findings of the work of Zachariah et al. who consider non-cardiovertible cardiac rhythms to be an important clue to an intracranial cause of CA (28, 29). Another interesting finding of our study is the disproportionate incidence of posterior circulation aneurysms in patients with OHCA. In comparison, aneurysms of the posterior circulation occurred in only 15% in our overall cohort, which is consistent with data from other studies. Previous studies on this topic report divergent results on aneurysm localization (30, 31). In our view, this is an abnormality that may well be related to the CA of these patients and could be a possible risk factor for this complication.

Nevertheless, immediate CPR is considered crucial to ensure survival after CA, which is why special attention is paid to lay CPR (17, 18, 32). Assuming that the “no-flow” time and thus organ perfusion should be interrupted as shortly as possible, this is considered crucial to increase the chance of primary survival. In connection with the time to onset of CPR, other factors such as the location (private home vs. busy public place) may play a role and thus potentially influence the outcome. However, the exact contextual conditions cannot be reconstructed and were therefore not evaluated in our retrospective study. The current American Heart Association (AHA) resuscitation guideline reports a 39% rate of patients receiving lay CPR. In our cohort, immediate lay CPR was performed in 52.6% of patients, which is consistent with these results. Patients without lay CPR had a significantly longer time to initiation of CPR, which is within comparable limits to other studies (17, 18, 33). Interestingly, this difference does not map in terms of ROSC, which was more likely to occur in professionally resuscitated than in lay resuscitated patients. A possible explanation for this is the presence of optimal equipment (defibrillator, ventilator, etc.) and the training of the emergency service personnel. That these points play an important role is repeatedly emphasized in all guidelines and studies on this topic (34, 35). However, no advantage in terms of initial survival could be shown in our study. The fact that in our cohort the type of CPR had no significant influence on survival could be due to the severe nature of SAH with frequently negative outcome and the small number of cases in our study. Overall, one-third of our patients survived SAH with OHCA and were discharged from the hospital. The rate of survivors in our cohort is thus higher than previously reported overall survival rates of 2% and 18% (10, 23).

After ROSC, the next step is expeditious transport to an appropriate hospital for further diagnosis and therapy (19, 36). Since in most cases a cardiac cause for the CA is assumed, hospitalization with a specialized intensive care unit and the possibility of coronary intervention is usually the goal (19). In our patient cohort, about half of the patients were treated initially according to this procedure, without a neurological cause being suspected. This resulted in a significantly, threefold longer duration to the first CCT scan compared to patients in whom a neurological cause was initially suspected. Early diagnosis plays a critical role in patients with SAH, and the consequence from a time delay is a loss of reasonable therapy options. Accordingly, we observed better survival rates with shorter duration to the CCT scan, which did not reach statistical significance (*p* = 0.065), most likely due to the small cohort size.

Despite the fact that we performed a bi-centric study and thus were able to screen more than 1,000 patients with SAH, the number of patients finally included in the study according to the inclusion criteria is low with 38 patients. Although our reported incidence is in accordance with the values commonly found in the literature, results have to be interpreted considering the limitation of a small case number. The Fisher scale was used to describe the severity of SAH. However, we cannot exclude the possibility that rebleeding may have occurred in the period between cardiac arrest and the acquisition of the CCT scan. Therefore, the Fisher score from the first CCT scan may not accurately reflect the severity of bleeding at the time of cardiac arrest in all patients. Another point to be noted is that the data on OHCA with CPR and the ER management were partly incomplete and not homogeneous. This ultimately led to the exclusion of seven patients from the final analysis. Moreover, we cannot exclude that the time to CCT scan was prolonged in unstable patients, which could represent a confounder. In addition, as a retrospective study, we do not provide long-term follow-up data and therefore cannot make any prospective statements.

## Conclusion

SAH is a relevant cause of OHCA. In pre-hospital care, immediate initiation of high-quality CPR is critical. Furthermore, a rapid diagnosis of SAH is crucial to achieve the best possible outcome. We therefore suggest that a CCT scan should be implemented in the treatment path for patients with ROSC after OHCA.

## Data Availability

The original contributions presented in the study are included in the article, further inquiries can be directed to the corresponding author.
